# Parliament and Publications

**Published:** 1910-09-24

**Authors:** 


### Parliament and Publications.

### SANITARY MEASURES IN INDIA.

An interesting Report on Sanitary Measures in India in 1908-1909 has been prepared for presentation to Parliament. It deals with the health of the European and native troops, of prisoners in gaols, and of the general population; also with vaccination, medical institutions (including hospitals), dispensaries, and medical schools, with lunatic asylums, and with sanitation.—"East India (Sanitary Measures)." Cd. 5245. Price 1s. 6d.

### LUNACY IN IRELAND.

The annual report of the Inspectors of Lunatics in Ireland shows that the number of insane on January 1, 1910, was 24,144, an increase on the year of 213. The number of the insane under care has increased from 250 per 100,000 of the population in 1880 to 552 per 100,000 in 1909. The rate of increase for the past four years has been five per annum, whereas the average during the entire period was over ten per annum. The percentage of recoveries on admission to asylums during 1909 was 38.4.—"Lunacy—Ireland." Cd. 5280. Price 1s.

### LUNACY IN SCOTLAND.

The fifty-second annual report of the General Board of Commissioners in Lunacy for Scotland states that on January 1 of the present year, exclusive of insane persons maintained at home, there were in Scotland 18,337 insane persons, including the inmates of training schools for imbecile children. Of these 2,560 were maintained from private sources, 15,724 by parochial rates, and 53 at the expense of the State. The increase since January 1, 1909, is 14.0. Although the number of patients has increased during the past five years the increase is greatly below that shown by any quinquennial period during the previous twenty-five years. The occurrence of insanity in proportion to population shows a marked decrease throughout the past five years. The number of patients removed from the register by discharge as recovered or unrecovered has been falling continuously during the past thirty years, and never to so large an extent as during the past five years.—"Fifty-Second Annual Report of the General Board of Commissioners of Lunacy in Scotland." Cd. 5315. Price 1s.

### BIRTHS, DEATHS, AND MARRIAGES IN IRELAND.

The annual report of the Registrar-General for Ireland, which deals with the year 1909, shows that the marriage rate was 5.18 per 1,000 of the estimated population, a decrease of 0.02, as compared with that for the year 1908; the birth-rate was 23.5 per 1,000 of the estimated population, being 0.2 above that for the preceding year; the death-rate (17.2 per 1,000) was 0.4 below the rate for the preceding year. In comparison with 1908, the deaths from all forms of tuberculous disease show a decrease of 699, this following a decrease in the year 1908 of 386, when compared with the year 1907. The Registrar-General states that this decrease is largely attributed to the work of the Countess of Aberdeen as carried on by the Women's National Health Association. The number of deaths from all forms of tuberculous disease during 1909 was 10,594, compared with 11,293 in 1908, and 12,135, the average for the ten years, 1899-1908. Among the so-called principal epidemic diseases the mortality in the year 1909 was increased for scarlet fever, whooping cough, and in a small degree for diphtheria, but for measles, typhus, enteric fever, and diarrhœal diseases there were decreases in mortality. There was a decrease in the mortality from influenza of 495 deaths, as compared with the year 1908. The figures for malignant diseases show an increase from 3,314 deaths in 1908 to 3,502 deaths in 1909. The infant mortality rate per 1,000 births was 92. In England and Wales for the year 1909 the infant mortality rate per 1,000 births registered was 109, and in Scotland for the year 1908 the rate was 121. These figures show that Ireland compares favourably in this respect with the sister countries. A comparison of the results, however, as regards the infant mortality in the three countries shows that Ireland generally occupies a rather unfavourable position. "Forty-sixth Detailed Annual Report of the Registrar-General for Ireland." Cd. 5265. Price 2s. 8d.

